# General and Specific Dimensions of Mood Symptoms Are Associated With Impairments in Common Executive Function in Adolescence and Young Adulthood

**DOI:** 10.3389/fnhum.2022.838645

**Published:** 2022-04-14

**Authors:** Elena C. Peterson, Hannah R. Snyder, Chiara Neilson, Benjamin M. Rosenberg, Christina M. Hough, Christina F. Sandman, Leoneh Ohanian, Samantha Garcia, Juliana Kotz, Jamie Finegan, Caitlin A. Ryan, Abena Gyimah, Sophia Sileo, David J. Miklowitz, Naomi P. Friedman, Roselinde H. Kaiser

**Affiliations:** ^1^Department of Psychology & Neuroscience, University of Colorado Boulder, Boulder, CO, United States; ^2^Department of Psychology, Brandeis University, Waltham, MA, United States; ^3^Department of Psychology, University of California, Los Angeles, Los Angeles, CA, United States; ^4^Department of Psychiatry, University of California, Los Angeles, Los Angeles, CA, United States

**Keywords:** depression, mood, bifactor, anxiety, anhedonia, mania, executive function, common EF

## Abstract

Both unipolar and bipolar depression have been linked with impairments in executive functioning (EF). In particular, mood symptom severity is associated with differences in common EF, a latent measure of general EF abilities. The relationship between mood disorders and EF is particularly salient in adolescence and young adulthood when the ongoing development of EF intersects with a higher risk of mood disorder onset. However, it remains unclear if common EF impairments have associations with specific symptom dimensions of mood pathology such as blunted positive affect, mood instability, or physiological arousal, or if differences in common EF more broadly relate to what is shared across various symptom domains, such as general negative affect or distress. To address this question, bifactor models can be applied to simultaneously examine the shared and unique contributions of particular mood symptom dimensions. However, no studies to our knowledge have examined bifactor models of mood symptoms in relation to measures of common EF. This study examined associations between common EF and general vs. specific symptom dimensions (anhedonia, physiological arousal, and mania) using structural equation modeling in adolescents and young adults with varying severity of mood symptoms (*n* = 495, ages = 13–25 years, 68.69% female). A General Depression factor capturing shared variance across symptoms statistically predicted lower Common EF. Additionally, a factor specific to physiological arousal was associated with lower Common EF. Anhedonia-specific and Mania-specific factors were not significantly related to Common EF. Altogether, these results indicate that deficits in common EF are driven by, or reflect, general features of mood pathology that are shared across symptom dimensions but are also specifically associated with physiological arousal.

## Introduction

Mood disorders, including depressive disorders and bipolar disorders, are common and impairing psychiatric conditions (Merikangas et al., 2010; Kessler et al., 2012; Walker et al., 2015). Mood disorders have been linked with cognitive impairments, which may contribute to worse functioning in daily life (Baune et al., 2010; Cotrena et al., 2016). In particular, mood disorders often present with impairments in executive function (EF; Snyder, 2013; Cotrena et al., 2016, 2020), the ability to plan and carry out goal-directed behavior. Mood disorders often emerge during adolescence and early adulthood (Paus et al., 2008), which also marks a time of considerable EF development (Best and Miller, 2010). A better understanding of the relationship between mood disorders and EF during this sensitive period may help us develop more effective early interventions to target cognitive impairments and improve functioning in daily life.

However, the nature of the association between mood disorders and EF remains unclear, in part due to the challenges of measuring these constructs. First, EF poses measurement challenges because EF involves controlling lower-level processes to carry out task-specific goals (Burgess, 1997; Miyake et al., 2000). Therefore, measures based on a single task can be contaminated by the specific lower-level processes required by the task. Second, EF is not a unitary construct, and tasks may vary in the extent to which they tap specific EFs (Miyake et al., 2000; Miyake and Friedman, 2012). For example, the Unity and Diversity model of EFs proposes that EFs can be broken down into shared and specific factors (Miyake et al., 2000; Miyake and Friedman, 2012). Common EF, which represents the variance shared across diverse EF tasks, has been theorized to relate to the general ability to develop and maintain a goal, and use that goal to direct ongoing behavior (Miyake and Friedman, 2012; Friedman and Miyake, 2017). However, performance on an individual task not only reflects common EF but also specific EFs (e.g., shifting between task sets, or updating the contents of working memory) and lower level perceptual processes (Miyake et al., 2000; Miyake and Friedman, 2012; Friedman and Miyake, 2017). Therefore, studies that only use a single task to measure EF face issues with task impurity and potential biases towards specific EFs.

Multi-task approaches combined with factor analysis can aid in addressing these issues in measuring EF by allowing for the separation of common and specific dimensions of EF and reducing task-specific impurities (Friedman and Miyake, 2017). Indeed, prior research adopting a latent variable approach to measuring EF has suggested that deficits in common EF correlate with higher severity of internalizing symptoms, including depressed mood as well as anxiety (Hatoum et al., 2018; Snyder et al., 2019). In contrast, specific EFs such as updating and shifting abilities have not been consistently linked to internalizing psychopathology (Hatoum et al., 2018; Snyder et al., 2019). Lower common EF may have significant potential to cause problems in everyday life due to its involvement in goal-directed behavior, which could include ignoring distracting thoughts, regulating emotions, and planning everyday tasks (Snyder et al., 2019). Other studies not explicitly deriving a common EF factor have found links between mood disorders and worse performance across a variety of EF tasks, which aligns with the hypothesis that mood pathology relates to what is shared across various EF tasks (for reviews, see Snyder, 2013; Cotrena et al., 2020). In sum, mood disorders appear broadly associated with deficits in common EF, reflecting general deficits in goal-directed behavior.

Whereas prior work has distinguished between common vs. specific dimensions of EF, it has tended to treat mood symptoms as unitary, ignoring the marked heterogeneity of symptoms that can characterize any individual’s clinical profile. Mood disorders are highly heterogeneous categories. Based on criteria from the *Diagnostic and Statistical Manual of Mental Disorders* (5th ed.; American Psychiatric Association, 2013), 227 unique symptom profiles of depression exist, and two individuals with that diagnosis may not have any shared symptoms. For example, some individuals diagnosed with a mood disorder display higher levels of anhedonia, defined as the loss of interest or pleasure (Pizzagalli, 2014). Others may report elevated physiological arousal or anxiety (Ionescu et al., 2013), while others may experience manic or hypomanic symptoms even if they do not meet the criteria for a bipolar diagnosis (McIntyre et al., 2017). Many psychiatric symptoms are also transdiagnostic, and appear across a range of disorders and at subthreshold levels in the general population (Dalgleish et al., 2020). It is unknown which of these symptom dimensions are associated with common EF, yet answering this question may help to explain why some individuals diagnosed with mood disorders experience higher levels of cognitive impairment.

However, investigating these issues requires facing a critical measurement challenge associated with the study of psychopathology: symptoms tend to be highly correlated (Caspi et al., 2014). Thus, even when studies have employed continuous measures of symptom dimensions, it remains ambiguous whether correlations are driven by unique attributes of that symptom dimension. The shared variance across symptom dimensions may itself represent a meaningful dimension of psychopathology that can help explain the association between mood disorders and EF (Caspi et al., 2014; Hatoum et al., 2018; Snyder et al., 2019). For example, the shared variance might reflect greater distress, general negative affect, or symptom burden. Deficits in EF may influence or reflect an individual’s ability to regulate their emotions (Zelazo and Cunningham, 2007; Pruessner et al., 2020), thereby increasing an individual’s susceptibility to experiencing greater distress or multiple types of mood symptoms (Joormann and Stanton, 2016). Lower common EF has also been linked with increased dependent stress (i.e., stressful events that an individual has some influence over) and subsequent rumination, which in turn may lead to a variety of mood symptoms (Snyder and Hankin, 2016; Snyder et al., 2019). In the opposite causal direction, a greater symptom burden may impair EF. For example, individuals experiencing increased symptom load may be more prone to negative, self-focused patterns of thought (Calvete et al., 2015), which may result in worse performance on measures of EF by consuming attentional resources (Watkins and Brown, 2002). However, to our knowledge, no studies have simultaneously examined how shared and specific mood symptom dimensions may relate to common EF. Therefore, the question remains: are associations between mood disorders and common EF driven primarily by what is shared across mood symptoms, or do certain symptom dimensions have additional unique associations with common EF?

To help address this question, bifactor models can serve as a means of simultaneously examining the shared and specific features of psychopathology symptoms. Bifactor models are a class of structural equation models that can partition a set of measures into shared and specific variance. A general factor captures the shared variance across all dimensions, and orthogonal specific factors capture variance unique to a particular dimension that remains after taking into account the general factor. We note that bifactor models have also come under critique; for example, these models can result in irregular or inadmissible results such as small or negative factor variances or loadings (Eid et al., 2017). Bifactor S-1 models have been proposed as a means of mitigating these issues (Eid et al., 2017; Burke and Johnston, 2020; Burns et al., 2020; Heinrich et al., 2020). These models use an additional set of indicators as a reference domain, which loads exclusively onto the general factor but does not have its own specific factor. Together, bifactor models have value as tools for partitioning variance, and bifactor S-1 models may help further address technical issues typically associated with bifactor models.

The current study used a bifactor S-1 model to examine associations between common EF and general and specific dimensions of mood symptoms in a sample of adolescents and young adults (ages 13–25 years) with high variance in symptoms of mood disorders. We chose to focus on this age group as it marks a critical developmental window that captures the intersection of EF development (Best and Miller, 2010; Friedman et al., 2016) and higher risk of emerging mood pathology (Paus et al., 2008). We pooled the sample across two study sites and study protocols to maximize the power and generalizability of the analyses. In confirmatory factor analyses, we examined associations between a common EF factor and a general depression factor representing shared variance across mood symptom dimensions, as well as specific factors anchored to items from canonical Anxious Arousal, Loss of Interest, Loss of Pleasure, and Mania scales. These symptom dimensions are prevalent and separable symptoms of psychopathology that often characterize or correspond with depressed mood (Watson et al., 1995a, b; Watson and Naragon-Gainey, 2010; Kendler, 2016). While anxious arousal is commonly associated with anxiety disorders (e.g., panic disorder) we note that altered physiological arousal and heightened anxiety have historically been considered central features of mood pathology (Kendler, 2016, 2017). Anxious arousal has also been previously used to predict cognitive functioning in individuals with depression (Quinn et al., 2012). Additionally, we evaluated exploratory bifactor models to compare data-driven symptom factors and their associations with Common EF against the confirmatory model. Based on prior research, we hypothesized that lower Common EF would be significantly associated with higher levels of the general depression factor from the confirmatory symptom model, and a similar general factor from the exploratory symptom model. Secondary analyses included tests of relationships between Common EF and each symptom dimension in its own model, to identify potential differences in results when shared symptom variance remained in the model.

## Materials and Methods

### Sample Characteristics

The sample consisted of 495 participants aged 13–25 years (*M* = 19.91, *SD* = 1.97; Table 1). 68.69% of the sample identified as female, 29.90% as male, and 1.41% as nonbinary, transgender, or other. The sample included subjects recruited for two research protocols with overlapping procedures. Both studies recruited participants from the Los Angeles and Boulder/Denver metropolitan areas. The present study pooled across these sites and studies to enhance statistical power, variance in mood symptoms, and generalizability. To be eligible for either study, participants had to speak fluent English, have normal vision, and have no reported head injuries or neurological conditions that might affect cognitive testing. Study 1 (*n* = 272) consisted of students recruited from the University of California Los Angeles (UCLA) and University of Colorado (CU) Boulder psychology subject pools (*n* = 213 from UCLA and *n* = 58 from CU Boulder). Study 2 (*n* = 223) consisted of individuals with current mood disorders at the time of recruitment, or individuals with no history of psychopathology (see Table 2). Individuals with mood disorder diagnoses (including Major Depressive Disorder, Persistent Depressive Disorder, Schizoaffective Disorder, Bipolar Disorder I, Bipolar Disorder II, and Bipolar NOS) were in depressive, dysthymic, or mixed episodes at the time of testing. Participants for Study 2 were recruited from the community and local clinics (*n* = 155 from the Los Angeles metropolitan area and *n* = 69 from Boulder/Denver metropolitan areas). The research protocols for these studies were approved by the Institutional Review Boards at UCLA and CU Boulder. Consent was obtained from legal adults (ages 18 and older) and parental consent and child assent were obtained from legal minors (ages 17 and younger) to participate in the study.

**Table 1:** Demographic characteristics of sample.

	Study 1		Study 2		Total
Total *n* = 495	UCLA	CU Boulder	UCLA	CU Boulder	
	**Mean (SD)**				
Age, years	20.22 (1.49)	19.31 (1.43)	20.33 (2.22)	18.51 (2.30)	19.91 (1.97)
	% of *n* = 213	% of *n* = 58	% of *n* = 155	% of *n* = 69	% of *n* =495
**Gender**				
Female	74.18%	65.52%	69.03%	53.62%	68.69%
Male	25.82%	34.48%	29.03%	40.58%	29.90%
Nonbinary/transgender/other	0%	0%	1.94%	5.80%	1.41%
**Race**					
African American	4.23%	5.17%	3.23%	1.45%	3.64%
American Indian/Alaskan native	0.94%	0%	0%	0%	0.40%
Asian	36.62%	3.45%	20.00%	7.25%	23.43%
Biracial or other	27.70%	5.17%	25.16%	13.04%	22.22%
Native Hawaiian/other Pacific Islander	0.47%	0%	0%	0%	0.20%
White	30.05%	86.21%	51.61%	78.26%	50.10%
**Ethnicity**				
Hispanic	22.07%	5.17%	20.65%	20.29%	19.39%
Not Hispanic or other	77.93%	94.83%	79.35%	79.71%	80.61%
**Education (Parent Highest)**					
Without high school diploma	9.39%	0%	8.39%	1.45%	6.87%
High school graduate without college degree	13.62%	3.45%	7.10%	1.45%	8.69%
Some college education	9.86%	10.34%	11.61%	15.94%	11.31%
Degree from four-year college	27.23%	32.76%	32.90%	26.09%	29.49%
Masters or other advanced degree	39.91%	53.45%	40.00%	53.62%	43.43%
Unknown	0%	0%	0%	1.45%	0.20%
**Annual Family Income**				
<10,000	5.63%	10.34%	10.97%	7.25%	8.08%
~10,000–25,000	8.37%	8.62%	9.68%	10.14%	9.09%
~25,000–50,000	13.15%	12.07%	12.90%	13.04%	12.93%
~50,000–75,000	17.84%	3.45%	16.13%	24.64%	16.57%
~75,000–100,000	17.84%	10.34%	14.84%	18.84%	16.16%
>100,000	37.09%	55.17%	34.84%	24.64%	36.77%
Unknown	0%	0%	0.65%	1.45%	0.40%

**Table 2:** Lifetime diagnoses of participants in Study 2, which consisted of individuals with current mood disorders at the time of recruitment, or individuals with no history of psychopathology.

Lifetime diagnoses	% of *n* = 223
**Mood disorders**	
Major Depressive Disorder	28.70%
Persistent Depressive Disorder	21.08%
Schizoaffective Disorder	0.90%
Bipolar I Disorder	4.48%
Bipolar II Disorder	3.59%
Bipolar NOS	2.24%
**Disorders secondary to mood disorders**
Anxiety disorders	30.94%
Agoraphobia	0.90%
Generalized Anxiety Disorder	18.83%
Panic Disorder	8.97%
Social Anxiety Disorder	12.56%
Specific Phobia	3.14%
Substance use disorders	23.32%
Mild	12.11%
Moderate	3.59%
Severe	7.62%
Eating disorders	8.52%
Anorexia Nervosa	3.14%
Bulimia Nervosa	4.04%
Binge Eating Disorder	0.45%
Eating Disorder NOS	0.90%
Attention-deficit/hyperactivity disorder	11.21%
Obsessive-compulsive disorder	3.14%
Post-traumatic stress disorder	16.14%

Diagnostic information was not collected in Study 1.

### Procedures

Participants were recruited for an in-person research session that included cognitive tasks followed by surveys. To enhance motivation, participants were informed that they would receive a monetary bonus based on their task performance (Participants did not know the specific bonus amount, and did not receive the bonus until the end of the research session). In Study 2, psychiatric history was evaluated after behavioral testing by a member of the research team using the Structured Clinical Interview for the DSM-5 (SCID; First et al., 2015; Table 2). Research staff (highly trained professional research assistants or graduate students) were trained in clinical evaluation by the principal investigator and attended weekly case consultation to review diagnoses and achieve inter-rater consensus. Additional procedures not relevant to the present analyses will be reported elsewhere.

### Executive Function (EF) Tasks

Executive functioning was measured using three well-validated tasks that have been previously used in latent variable analyses, with a variety of samples including adolescents and young adults (for review, see Friedman and Miyake, 2017). These tasks were adapted from Friedman et al. (2016). These tasks have previously demonstrated good internal reliability and load onto a common EF factor (e.g., Friedman et al., 2008, 2011, 2016). These three tasks were chosen because they differ in their perceptual characteristics and demands on specific subdimensions of EF, and hence their shared variance should primarily reflect shared processes across goal-directed EF tasks.

#### Antisaccade Task

**Figure 1:**
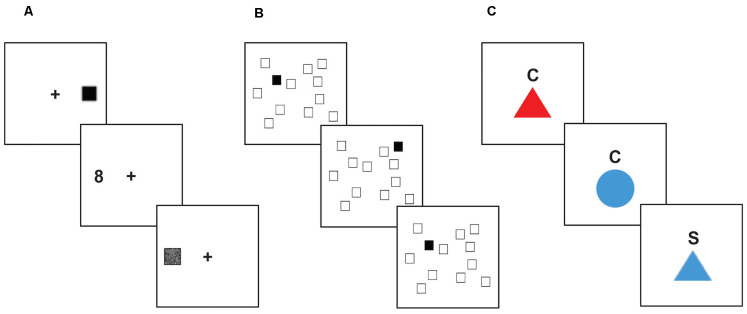
Executive function tasks. Stimuli shown are not to scale. **(A)** *Antisaccade task*. For each trial, the participant focused on a fixation point at the center of the screen. After a variable amount of time, a small black square would appear on one side of the screen, quickly followed by a number (between 1 and 9) appearing on the opposite side of the screen. The participant was instructed to inhibit their saccade to the visual distractor, and instead quickly look to the target stimulus to identify it before it was covered up, and then report the number they saw to the research assistant. **(B)** *Spatial n-back task*. In each block of this task, the participant saw a display of twelve white squares distributed across the screen. On each trial, one square in the display turned black. The participant was instructed to respond by pressing a button to indicate whether the black square appeared in the same spatial location as the black square two trials earlier. **(C)** *Color-shape task*. On each trial, a stimulus appeared in the center of the screen below a cue letter (C or S) indicating whether the participant should categorize the shape based on color (red or blue) or shape (circle or triangle). Trial types were interspersed such that participants were required to assess on each trial whether to use the same categorization rule as the previous trial (“repeat” trials), or if they needed to switch to the other categorization rule (“switch” trials).

This task is a measure of response inhibition (Figure 1A). Participants were instructed to control eye movements (saccades) in order to read numbers that briefly appeared on the screen. Participants were told to focus on a fixation cross in the middle of the screen. After a variable amount of time (ranging from 1,500 to 3,500 ms), a small black square (1/16 inch) flashed on either the left or right side of the screen for 150 ms (3.75 inches away from the fixation cross). Afterward, a number (1–9) in a box (3/8 inch) appeared on the opposite side of the screen but was quickly covered up by a gray box. In order to read the number in the box, participants needed to prevent themselves from looking at the initial black square and look at the opposite side of the screen to see the number. Participants said the number aloud and a researcher recorded their answers. Each participant first completed 36 trials in which the distractor square and the target appeared on the same side (prosaccade trials) to acquaint them with the task and reinforce the prepotent prosaccade response, followed by 12 practice trials in which the target appeared on the opposite side of the screen (antisaccade trials). The participant then completed three blocks (24 trials each) of antisaccade trials. The first, second, and third blocks showed the cue for 250, 233, and 200 ms respectively for progressively increasing difficulty. The dependent measure was accuracy across the three antisaccade blocks.

#### Spatial N-Back Task

This task is a measure of working memory updating (Figure 1B). Participants were shown a screen which displayed 12 scattered white squares. On each trial, one of the squares turned black for 500 ms, followed by an inter-trial interval of 1,500 ms. Participants responded *via* a button box whether or not each black square represented a match. When the black square appeared in the same location as it was two trials previously, that indicated a “match”. Within each block of trials, 25% of the trials were match trials. Participants started with a practice block of 20 trials. The task consisted of five blocks, 24 trials each. The dependent measure was overall accuracy.

#### Color-Shape Task

This task is a measure of shifting ability (Figure 1C). On a computer monitor, participants were presented with a series of circles or triangles that were colored red or blue. A letter appeared above the shape on each trial: “C” for color, or “S” for shape. When the letter “C” was above the shape, participants were instructed to indicate using a button box whether the shape was red or blue. When the letter “S” was above the shape, participants responded using the button box to indicate whether the shape was a circle or triangle. Trials that switched to a color categorization after a shape categorization trial, or vice-versa, were switch trials; trials that required sequential color (or shape) categorization were non-switch trials. Participants were given 12 trials to practice color identification, 12 trials to practice shape categorization, and 24 practice trials that included intermixed color and shape identification trials. The task consisted of two blocks of 52 trials each, with mixed color and shape trials. Half of the mixed trials were switch trials. The dependent measure was the switch cost, measured as the difference in reaction time between switch and non-switch trials.

### Symptom Measures

Participants completed the general distress—depression, anxious arousal, loss of interest, and loss of pleasure subscales of the mood and anxiety symptom questionnaire (MASQ; Watson et al., 1995a, b; Table 3). This measure has been shown to have high reliability and validity (Watson et al., 1995a, b). For each item, participants were asked to indicate the extent to which they experienced various symptoms in the past week, on a Likert scale from 1 (not at all) to 5 (extremely). The 12-item general distress—depression subscale included statements such as “Felt hopeless.” The 17-item anxious arousal subscale included statements like, “Heart was racing or pounding.” The 8-item loss of interest subscale consisted of statements such as “Felt like it took an extra effort to get started.” The 14-item loss of pleasure subscale included reverse-scored statements such as “Felt really happy.” See Table 3 for lists of all items.

**Table 3:** Items from the MASQ and GBI scales.

**MASQ Scales**	**GBI Scale**
**MASQ General Distress—Depression**	**GBI Mania**
1. Felt sad 2. Felt discouraged 3. Felt worthless 4. Felt depressed 5. Felt like a failure 6. Blamed myself for a lot of things 7. Felt inferior to others 8. Felt like crying 9. Was disappointed in myself 10. Felt hopeless 11. Felt sluggish or tired 12. Felt pessimistic about the future	1. Feeling depressed or irritable, OR feeling extremely high, elated, and overflowing with energy? 2. Feeling unusually happy and intensely energetic, but also feeling like almost everything gets on your nerves and makes you irritable or angry? 3. Feeling like your mood or energy shifts rapidly back and forth from happy to sad or high to low? 4. Finding that your feelings or energy are generally up or down, but rarely in the middle? 5. Experiencing periods when, although you are feeling unusually happy and intensely energetic, you are also physically restless, unable to sit still, and have to keep moving or jumping from one activity to another? 6. Having periods of extreme happiness and intense energy when you also feel much more anxious or tense (jittery, nervous, uptight) than usual? 7. Having periods when your friends or other family members told you that you seemed unusually happy or high—clearly different from your usual self or from a typical good mood? 8. Having times when your thoughts and ideas came so fast that you couldn’t get them all out, or they came so quickly others complained that they couldn’t keep up with your ideas? 9. Having times when, although you were feeling unusually happy and intensely energetic, you also had to struggle very hard to control inner feelings of rage or an urge to smash or destroy things? 10. Feeling extremely happy and intensely energetic and it took you more than an hour to get to sleep at night?
**MASQ Loss of Interest**
1. Felt unattractive 2. Felt withdrawn from other people 3. Felt really slowed down 4. Felt really bored 5. Felt like it took an extra effort to get started 6. Felt like nothing was very enjoyable 7. Felt like there wasn’t anything interesting or fun to do 8. Thought about death or suicide	
**MASQ Anxious Arousal**
1. Startled easily 2. Hands were shaky 3. Was short of breath 4. Felt faint 5. Had hot or cold spells 6. Hands were cold or sweaty 7. Was trembling or shaking 8. Had trouble swallowing 9. Felt dizzy or lightheaded 10. Had a pain in my chest 11. Felt like I was choking 12. Muscles twitched or trembled 13. Had a very dry mouth 14. Was afraid I was going to die 15. Heart was racing or pounding 16. Felt numbness or tingling in my body 17. Had to urinate frequently
**MASQ Loss of Pleasure**
1. Felt cheerful 2. Felt optimistic 3. Felt really happy 4. Was proud of myself 5. Felt like I was having a lot of fun 6. Felt like I had a lot of energy 7. Felt really up or lively 8. Looked forward to things with enjoyment 9. Felt like I had a lot of interesting things to do 10. Felt like I had accomplished a lot 11. Felt like I had a lot to look forward to 12. Felt hopeful about the future 13. Seemed to move quickly and easily 14. Felt really good about myself

The general behavior inventory (GBI; Depue, 1987) 10-item mania subscale was used to assess manic symptoms^1^ (Table 3). This measure has been shown to have high reliability and validity (Youngstrom et al., 2021). This measure asked to what extent an individual experienced symptoms such as “Feeling unusually happy and intensely energetic, but also feeling like almost everything gets on your nerves and makes you irritable or angry.” Participants rated these questions on a Likert scale from 0 (never or hardly ever) to 3 (very often or almost constantly). See Table 3 for lists of all items.

### Statistical Analyses

#### Data Trimming and Checks

We used the same exclusions and trimming procedures used in prior studies using these EF tasks (e.g., Friedman et al., 2016, 2020). For the models including EF measures, scores on any of the EF tasks with chance-level accuracy were removed (22 task scores excluded). For the color-shape task, RTs on incorrect responses, RTs for trials immediately following incorrect responses, and RTs below 200 ms were removed. Then, observations >3.32 times the median absolute deviation in each condition were removed before calculating condition averages. This within-subject trimming procedure results in a better measure of central tendency compared to untrimmed averages, improving reliability (Wilcox and Keselman, 2003). See Tables s 4, 5 for bivariate correlations, descriptive statistics, and, reliability checks for symptom and task measures.

**Table 4:** Correlations between symptom measures (total scores) and task measures.

**Variable**	**Correlation**						
	Anx.	LOI	LOP	Gen.	Man.	CS	AS
Anxious Arousal	-	-	-	-	-	-	-
Loss of Interest	0.22	-	-	-	-	-	-
Loss of Pleasure	0.62	0.56	-	-	-	-	-
General Distress-Depression	0.63	0.57	0.85	-	-	-	-
Mania	0.57	0.18	0.48	0.48	-	-	-
CS switch-cost (ms)	0.06	−0.07	0.03	0.05	0.03	-	-
AS Accuracy	−0.17	−0.03	−0.11	−0.13	−0.12	−0.14	-
SB Accuracy	−0.17	−0.03	−0.04	−0.09	−0.11	−0.12	0.29

*Anx., Anxious Arousal; LOI, Loss of Interest; LOP, Loss of Pleasure; Gen., General Distress—Depression; Man., Mania; CS, Color-shape; AS, Antisaccade; SB, Spatial 2-back*.

**Table 5:** Descriptive statistics of model variables.

**Variable**	**Reliability**	**Mean**	**SD**
Anxious Arousal	0.89^a^	25.99	9.23
Loss of Interest	0.88^a^	17.45	7.09
Loss of Pleasure	0.95^a^	45.64	12.32
General Distress—Depression	0.95^a^	27.57	11.96
Mania	0.89^a^	3.91	4.86
CS switch-cost (ms)	0.93^b^	229.48	182.94
AS Accuracy	0.95^b^	0.654	0.17
SB Accuracy	0.98^b^	0.810	0.08

*^a^Internal reliability was calculated using Cronbach’s alpha. ^b^Internal reliability was calculated by adjusting split-half correlations with the Spearman-Brown prophecy formula*.

#### Model Estimation

We estimated structural equation models using Mplus version 8.1 (Muthén and Muthén, 1998-2017). To assess model fit, we examined χ^2^ values (a non-significant χ^2^ value indicates a good fit, although χ^2^ may remain significant for larger sample sizes despite acceptable fit according to other measures), the comparative fit index (CFI > 0.95 indicating good fit), and the standardized root-mean-square residual (SRMR < 0.08 indicating good fit; Hu and Bentler, 1998). Due to the inclusion of ordinal symptom measures, we used a robust diagonally weighted least squares estimator (WLSMV) for all models. The models used a probit link function to estimate an underlying normal liability for each symptom. For the confirmatory bifactor model, we also calculated statistical indices of factor reliability and validity (ω, ω^H^, ECV, H) that have been previously recommended for such models (Rodriguez et al., 2016). Standardized estimates are reported throughout. These standardized estimates are measures of effect size and range from −1 to 1, where the value represents the predicted change in standard deviations of the outcome variable for one standard deviation change in the predictor variable (Muthén and Muthén, 1998-2017; Kelley and Preacher, 2012). Following standard practices, we first evaluated the fit of the symptom measurement models, prior to testing the structural models (Kline, 2016). To test our primary hypotheses, we examined structural models assessing associations between common EF and symptom dimensions using confirmatory and exploratory bifactor symptom models. Secondary analyses included tests of associations between Common EF and each symptom dimension in its own model, to examine potential differences in results when shared symptom variance was not removed. We corrected for multiple comparisons using a false discovery rate (FDR) procedure (*q* < 0.05; Benjamini and Hochberg, 1995).

#### Site Checks

We tested the invariance of the confirmatory bifactor symptom model across sites (UCLA and CU Boulder). When comparing levels of invariance, changes in CFI less than or equal to 0.01 and changes in SRMR less than or equal to 0.015 were interpreted as acceptable changes in fit, since χ^2^ difference tests of invariance can still be significant with large samples even when the absolute differences in model estimates are marginal (Cheung and Rensvold, 2002; Chen, 2007; Meade et al., 2008). We tested models with configural invariance and scalar invariance, as recommended for models with ordinal variables (Muthén and Muthén, 1998-2017). Configural invariance tests whether the factor structure differs significantly between groups. The configural model had acceptable fit, *χ^2^*_(3456)_ = 5052.041, *p* < 0.001, CFI = 0.957, SRMR = 0.086. Scalar invariance tests whether the factor loadings and thresholds differ significantly between groups. The scalar model also had acceptable fit, *χ^2^*_(3713)_ = 5217.399, *p* < 0.001, CFI = 0.960, SRMR = 0.089, but had a significantly worse chi-square, Δ*χ^2^*_(257)_ = 379.35, *p* < 0.001. However, the changes in CFI and SRMR values were less than the cutoffs suggested by Chen (2007), indicating an acceptable level of scalar invariance across sites. Therefore, we proceeded to use a model with participants from both sites in a single group for subsequent analyses.

#### Measurement Models

##### EF Model

For the EF model, antisaccade accuracy, spatial 2-back accuracy, and color-shape switch-cost loaded on a Common EF factor. The color-shape switch-cost was converted to seconds so that its variance would be on a similar scale to the other measures. Additionally, switch-cost was multiplied by negative one so that for all three measures of EF, higher values indicate better performance.

##### Bifactor Symptom Model

We first tested a symptom measurement model with confirmatory factor analysis (CFA). In this bifactor (S-1) model, items from canonical scales for general distress—depression, anxious arousal, loss of interest, loss of pleasure, and mania symptoms loaded onto a general depression factor. Anxious arousal, loss of interest, loss of pleasure, and mania symptoms loaded onto specific factors orthogonal to one another.

##### Exploratory Models

Exploratory factor analysis (EFA) bifactor symptom models were tested, with the number of factors ranging from three to five (one general factor, and two to four specific factors). We used exploratory structural equation modeling (ESEM) in Mplus (Muthén and Muthén, 1998-2017) to specify these models, which enabled us to use the resulting EFA factors in subsequent structural equation models. EFA allows for a data-driven exploration of factor structure that imposes fewer restrictions than CFA, which constrains all cross-loadings to equal zero (Marsh et al., 2014). This approach can allow for a less biased representation of the data structure and inter-factor associations, particularly for item-level models where small but significant cross-loadings are likely (Marsh et al., 2014).

#### Structural Models

##### Bifactor Symptom Model Factors and Common EF

The Common EF factor was regressed on the orthogonal symptom factors to test associations between EF and general vs. specific dimensions of mood symptoms.

##### Standalone Symptom Factors and Common EF

Standalone symptom models were also tested, in which the Common EF factor was regressed on a single symptom factor in its own model, in order to compare results when shared variance across symptoms remained intact.

##### Exploratory Symptom Factors and Common EF

After selecting the best exploratory symptom model, the Common EF factor was regressed on the exploratory symptom factors to test associations between EF and general vs. specific dimensions of mood symptoms.

## Results

### Measurement Models

#### EF Model

**Figure 2:**
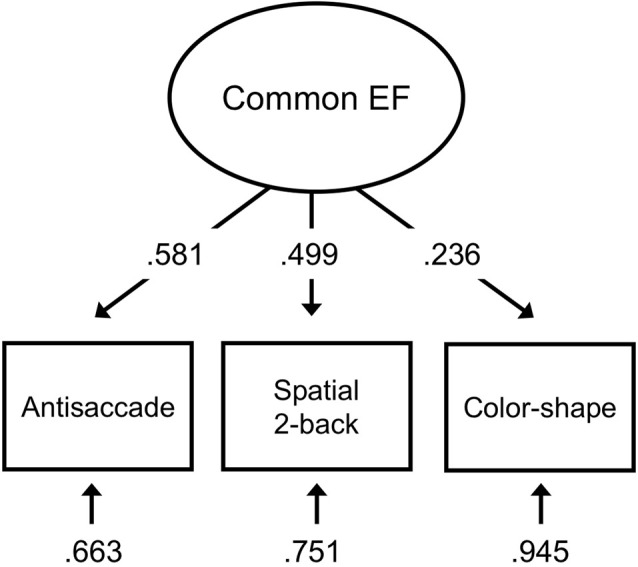
EF measurement model with antisaccade accuracy, spatial 2-back accuracy, and color-shape reaction time switch cost representing inhibition, updating, and shifting abilities, respectively. Standardized estimates shown.

The three EF task measures loaded significantly (*p*s < 0.05) onto the Common EF factor (Figure 2). The stand-alone model was saturated (had zero df), so perfectly fit the data.

#### Bifactor Symptom Model

**Figure 3:**
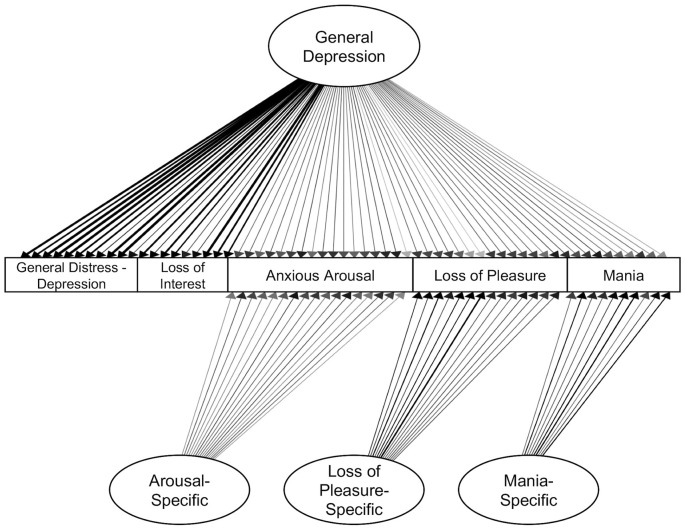
Confirmatory bifactor model of symptoms. Standardized estimates shown. All factor loadings were significant (*p*s < 0.05). Darker lines indicate larger loadings.

An initial bifactor S-1 model included specific factors for loss of interest, loss of pleasure, anxious arousal, and mania symptom dimensions as defined by canonical scales. The general distress—depression items were specified to only load on the general depression factor. The resulting model did not have a positive definite matrix. Inspection of the results suggested an issue with the loss of interest specific factor, which had several unexpectedly negative loadings and an unstandardized variance of zero. Therefore, we tested a model in which the specific factor for loss of interest was dropped (consistent with prior studies using similar items, e.g., Banich et al., 2020). This bifactor model included specific factors for loss of pleasure, arousal, and mania (Table 6, Figure 3). The loss of interest and general distress—depression items were specified to only load on the general depression factor. This model had adequate fit, *χ^2^*_(1728)_ = 3,738.310, *p* < 0.001, CFI = 0.948, SRMR = 0.076. All item loadings were significant (*p*s < 0.05). Inspection of modification indices suggested a significant amount of unexplained covariance remaining between the mania-specific and arousal-specific factors [modification index (MI) = 557.517]. However, this correlation was not added to keep the symptom factors orthogonal to one another.

**Table 6:** Item loadings in the confirmatory bifactor model.

**Items**	**Standardized loadings**	
	**General factor**	**Specific factor**
**MASQ General Distress—Depression**	
Gen. 1	0.854	
Gen. 2	0.794	
Gen. 3	0.894	
Gen. 4	0.908	
Gen. 5	0.885	
Gen. 6	0.810	
Gen. 7	0.805	
Gen. 8	0.690	
Gen. 9	0.876	
Gen. 10	0.903	
Gen. 11	0.641	
Gen. 12	0.815	
**MASQ Loss of Interest**	
LOI 1	0.687	
LOI 2	0.822	
LOI 3	0.763	
LOI 4	0.588	
LOI 5	0.654	
LOI 6	0.879	
LOI 7	0.816	
LOI 8	0.780	
**MASQ Anxious Arousal**		**Anxious Arousal**
Anx. 1	0.493	0.363
Anx. 2	0.540	0.575
Anx. 3	0.432	0.501
Anx. 4	0.532	0.433
Anx. 5	0.451	0.379
Anx. 6	0.517	0.451
Anx. 7	0.598	0.630
Anx. 8	0.402	0.488
Anx. 9	0.525	0.540
Anx. 10	0.551	0.440
Anx. 11	0.470	0.524
Anx. 12	0.420	0.616
Anx. 13	0.485	0.430
Anx. 14	0.517	0.368
Anx. 15	0.572	0.560
Anx. 16	0.595	0.515
Anx. 17	0.222	0.323
**MASQ Loss of Pleasure**		**Loss of Pleasure**
LOP 1	0.526	0.688
LOP 2	0.490	0.670
LOP 3	0.497	0.727
LOP 4	0.527	0.608
LOP 5	0.427	0.706
LOP 6	0.256	0.704
LOP 7	0.196	0.777
LOP 8	0.530	0.585
LOP 9	0.520	0.589
LOP 10	0.514	0.523
LOP 11	0.484	0.617
LOP 12	0.511	0.590
LOP 13	0.387	0.530
LOP 14	0.605	0.603
**GBI Mania**		**Mania**
Man. 1	0.564	0.519
Man. 2	0.476	0.713
Man. 3	0.534	0.590
Man. 4	0.610	0.562
Man. 5	0.477	0.658
Man. 6	0.422	0.717
Man. 7	0.353	0.745
**GBI Mania**		**Mania**
Man. 8	0.536	0.491
Man. 9	0.412	0.663
Man. 10	0.291	0.727

*All loadings were significant (*ps* < 0.05). Anx., Anxious Arousal; LOI, Loss of Interest; LOP, Loss of Pleasure; Gen., General Distress—Depression; Man., Mania. See Table 3 for item content*.

Omega reliability (ω) coefficients were high for all factors (ωs = 0.939 − 0.983). The Omega hierarchical (ω^H^) value, or the percent of total score variance explained by the general factor, was high for the General Depression factor (ω^H^ = 0.855). Omega hierarchical specific values (ω^HS^), or the percent of subscale score variance attributable to the corresponding specific factor, were moderate for the Arousal-specific factor (ω^HS^ = 0.459), Loss of Pleasure-specific factor (ω^HS^ = 0.629), and Mania-specific factor (ω^HS^ = 0.616). The Explained Common Variance (ECV), or the percent of variance captured by each factor out of all explained variance of a factor’s items, was 61.6% for the general depression factor, 48.9% for the arousal-specific factor, 64.6% for the loss of pleasure-specific factor and 64.6% for the mania-specific factor. While all symptom dimensions had both general and specific variance, loss of pleasure and mania had slightly more specific variance, while anxious arousal variance was somewhat better captured by the general factor. The *H* values, which indicate construct reliability and replicability, were above the minimum cutoff of 0.70 (Rodriguez et al., 2016) for all factors (*H*s = 0.848 − 0.983).

#### Exploratory Bifactor Models

Next, we tested exploratory bifactor models to identify general and specific symptom factors using a data-driven approach. This approach allows for a less restrictive exploration of factor structure compared with CFA and allows for items to load on multiple specific factors (Marsh et al., 2014). Bifactor symptom models with three to five factors were tested (one general factor plus two to four specific factors). The 5-factor model fit well, *χ^2^*_(1535)_ = 2,302.372, *p* < 0.001, CFI = 0.980, SRMR = 0.037, and fit significantly better than the 4-factor model, Δ*χ^2^*_(57)_ = 347.471, *p* < 0.001, which fit significantly better than the 3-factor model, Δ*χ^2^*_(58)_ = 493.001, *p* < 0.001.

**Figure 4:**
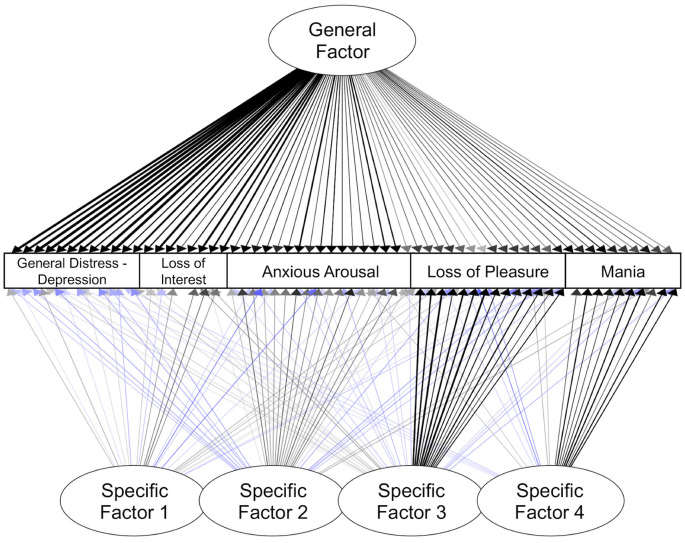
Exploratory bifactor model of symptoms. Standardized estimates shown. Only significant paths are shown here (*p*s < 0.05). Blue lines indicate negative loadings. Darker lines indicate loadings with a larger absolute value.

The 5-factor model included a general factor with significant loadings from all symptoms (Table 7, Figure 4). Similar to the confirmatory bifactor model, the items with the highest loadings on this factor came from the canonical general distress—depression and loss of interest scales.

**Table 7:** Item loadings in the 5-factor exploratory bifactor model.

**Items**	**Standardized loadings**				
	**G**	**S1**	**S2**	**S3**	**S4**
**MASQ General Distress—Depression**					
Gen. 1	**0.825**	0.187	−0.165		
Gen. 2	**0.781**	−0.086	−0.203	0.074	
Gen. 3	**0.866**		−0.233		
Gen. 4	**0.869**	0.222		0.143	
Gen. 5	**0.854**		−0.266	0.101	−0.089
Gen. 6	**0.824**	−0.102			
Gen. 7	**0.774**		−0.207	0.128	−0.107
Gen. 8	**0.695**	0.104			
Gen. 9	**0.855**		−0.255		−0.114
Gen. 10	**0.871**	0.117	−0.191	0.112	
Gen. 11	**0.656**	−0.205			−0.142
Gen. 12	**0.761**		−0.258	0.184	
**MASQ Loss of Interest**					
LOI 1	**0.670**			0.093	
LOI 2	**0.797**	0.151		0.114	
LOI 3	**0.766**	−0.161			
LOI 4	**0.559**	0.276			
LOI 5	**0.652**				
LOI 6	**0.785**	0.371		0.267	
LOI 7	**0.728**	**0.435**	0.084	0.224	
LOI 8	**0.748**	0.323			
**MASQ Anxious Arousal**					
Anx. 1	**0.575**		0.195	−0.122	
Anx. 2	**0.642**		**0.454**		
Anx. 3	**0.549**	−0.135	0.315	−0.161	
Anx. 4	**0.612**	−0.367	0.251		
Anx. 5	**0.507**	−0.136	0.289		
Anx. 6	**0.596**		0.324		
Anx. 7	**0.714**		**0.486**	−0.132	
Anx. 8	**0.513**		0.342	−0.171	
Anx. 9	**0.637**	−0.350	0.355	−0.098	
Anx. 10	**0.634**		0.273		
Anx. 11	**0.596**		0.322	−0.209	
Anx. 12	**0.535**		**0.513**		
Anx. 13	**0.566**	−0.119	0.261		0.167
Anx. 14	**0.593**		0.210	−0.130	
Anx. 15	**0.680**		0.375	−0.126	
Anx. 16	**0.675**		0.398		0.140
Anx. 17	0.316		0.187	−0.164	
**MASQ Loss of Pleasure**					
LOP 1	**0.456**	0.211		**0.720**	
LOP 2	**0.432**	0.078	−0.127	**0.696**	
LOP 3	**0.434**	0.192		**0.748**	−0.132
LOP 4	**0.475**		−0.241	**0.620**	
LOP 5	0.373	0.167		**0.720**	−0.164
LOP 6	0.245			**0.684**	−0.357
LOP 7	0.171			**0.751**	−0.252
LOP 8	**0.471**	0.081		**0.638**	
LOP 9	**0.463**	0.090		**0.635**	
LOP 10	**0.468**		−0.269	**0.543**	
LOP 11	**0.421**		−0.119	**0.672**	0.210
LOP 12	**0.448**	−0.100	−0.258	**0.629**	0.179
LOP 13	0.358	−0.172		**0.565**	
LOP 14	**0.554**		−0.171	**0.632**	
**GBI Mania**					
Man. 1	**0.594**				**0.473**
Man. 2	**0.519**				**0.658**
Man. 3	**0.559**				**0.575**
Man. 4	**0.632**				**0.538**
Man. 5	**0.525**			−0.121	**0.603**
Man. 6	**0.471**		0.187	−0.149	**0.662**
**GBI Mania**					
Man. 7	**0.422**			−0.216	**0.670**
Man. 8	**0.548**		0.234		**0.441**
Man. 9	**0.438**				**0.653**
Man. 10	0.369			−0.210	**0.639**

*Only significant loadings are listed here (*p*s < 0.05). Loadings > 0.4 are bolded. G, general factor; S1-S4, Specific Factor 1–Specific Factor 4; Anx., Anxious Arousal; LOI, Loss of Interest; LOP, Loss of Pleasure; Gen., General Distress—Depression; Man., Mania. See Table 3 for item content*.

Specific Factor 1 reflected anhedonic symptoms and sad mood. The items with the largest significant loadings on Specific Factor 1 came from the canonical loss of interest scale (e.g., “Felt like there wasn’t anything interesting or fun to do”). In addition, there were small but significant loadings from items reflecting sad mood, absence of fatigue, and low physiological arousal.

Specific Factor 2 reflected elevated physiological arousal and some items related to positive affect. The items with the largest loadings on Specific Factor 2 came from the canonical anxious arousal scale (e.g., “Muscles twitched or trembled” and “Hands were shaky”). All items from this scale had significant loadings on this factor. Specific Factor 2 also included small but significant loadings reflecting low negative affect and high positive affect.

Specific Factor 3 appeared to primarily capture low positive affect. The strongest indicators for Specific Factor 3 came from the canonical loss of pleasure scale (e.g., reverse-coded “Felt really up or lively”, and “Felt really happy”). All items from the loss of pleasure scale loaded significantly on this factor. More than half of the anxious arousal items also had small but significant negative loadings on this factor, reflecting low levels of physiological symptoms.

Specific Factor 4 primarily reflected symptoms of mania. The strongest indicators for Specific Factor 4 were from the mania scale (e.g., “Feeling unusually happy and intensely energetic, but also feeling like almost everything gets on your nerves and makes you irritable or angry”). All items from this scale loaded significantly on this factor. Other items that had small but significant loadings on Factor 4 included items reflecting higher levels of energy but lower levels of anticipatory pleasure.

Altogether, these exploratory analyses revealed a set of symptom factors with clear similarities to the CFA model (e.g., separability of specific factors reflecting heightened physiological arousal, low positive affect, and manic symptoms), as well as notable differences, such as the emergence of an additional anhedonia-like factor (Specific Factor 1).

### Structural Models

#### Confirmatory Bifactor Symptom Model Factors and Common EF

**Figure 5:**
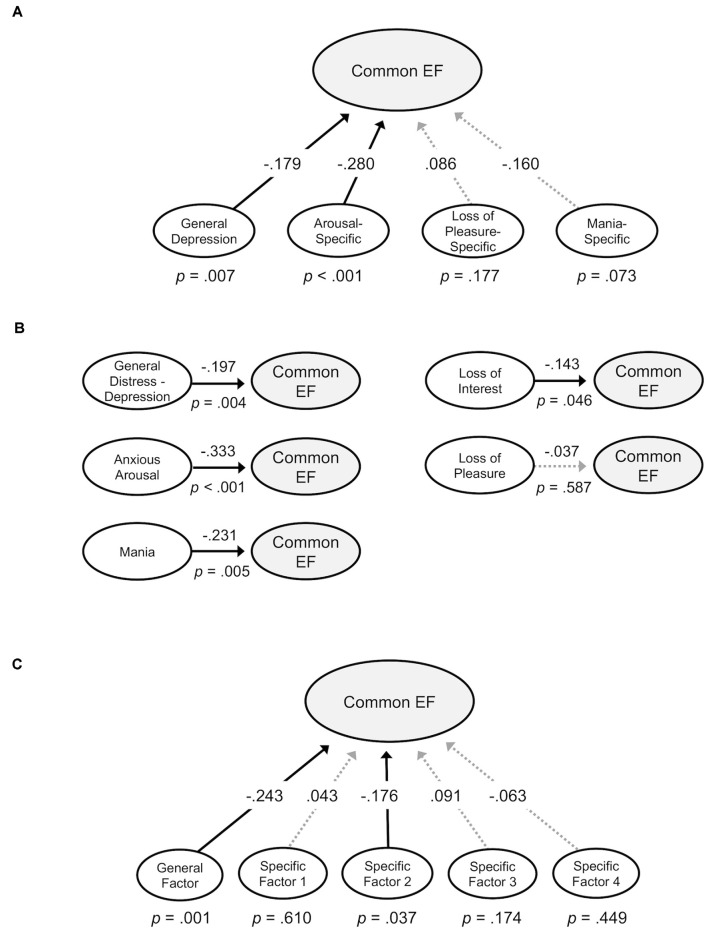
Structural models examining associations between Common EF and symptom factors. Standardized estimates shown. Significant paths are shown in solid lines; non-significant paths are shown in lighter dotted lines. **(A)** Common EF regressed on general depression, arousal-specific, loss of pleasure-specific, and mania-specific factors from a confirmatory bifactor model. **(B)** Common EF regressed on general distress—depression, anxious arousal, loss of pleasure, loss of interest, and mania factors from standalone models. **(C)** Common EF regressed on general and specific factors from an exploratory bifactor model.

Next, structural equation models were examined that evaluated the relationship between the common EF factor and the mood symptom factors derived in confirmatory factor analyses. The first model incorporated the bifactor symptom model and tested for significant effects of canonical general depression, loss of pleasure-specific, arousal-specific, and mania-specific factors on common EF (Figure 5A). This model had acceptable fit, *χ^2^*_(1907)_ = 3,877.865, *p* < 0.001, CFI = 0.950, SRMR = 0.074. General depression had a small but significant association with lower common EF, *β* = −0.179, *p* = 0.007. Additionally, the arousal-specific factor was significantly associated with lower common EF, *β* = −0.280, *p* < 0.001, with a small effect size. These effect sizes are comparable to those identified previously between psychopathology factors and EF measures (e.g., Caspi et al., 2014; Martel et al., 2017; Hatoum et al., 2018). Altogether, this model explained 14.4% of the common EF factor variance. There were no significant site differences in terms of associations between common EF and the various symptom dimensions (*p*s > 0.05). These effects remained significant after FDR corrections (*q*s < 0.05).

The same model was tested while controlling for the potential effects of age and gender on common EF. We included age as a predictor of the common EF factor, based on prior work showing changes in this general factor with age (Friedman et al., 2016). Gender was controlled at the EF indicator level, based on prior research showing gender differences at the levels of specific EF tasks, but not at the level of latent variables (Friedman et al., 2011, 2016). This model had acceptable fit, *χ^2^*_(2031)_ = 4,027.979, *p* < 0.001, CFI = 0.951, SRMR = 0.085. The main effects remained significant: greater General Depression significantly related to lower common EF, β = −0.198, *p* = 0.002, and the arousal-specific factor was also significantly associated with lower common EF, *β* = −0.283, *p* < 0.001. There was also a significant effect of gender on antisaccade accuracy, *β* = −0.219, *p* < 0.001, consistent with prior work finding males having higher accuracy on this task than females, on average (Friedman et al., 2016).

#### Standalone Symptom Factors and Common EF

Additional structural models were tested that evaluated the relationship between common EF and each symptom factor derived from confirmatory factor analysis in a separate model (Figure 5B). Since these models do not remove the shared variance across symptom dimensions, they provide complementary information about how particular dimensions might contribute to the association between general depression and common EF.

The first model tested the effect of the general distress—depression factor on common EF. This model had good fit, *χ^2^*_(89)_ = 292.796, *p* < 0.001, CFI = 0.989, SRMR = 0.030. The General distress—depression factor was significantly associated with lower common EF, *β* = −0.197, *p* = 0.004. The second model tested the effect of the anxious arousal factor on common EF. This model had good fit, *χ^2^*_(169)_ = 330.944, *p* < 0.001, CFI = 0.969, SRMR = 0.044. The anxious arousal factor was significantly associated with lower common EF, *β* = −0.333, *p* < 0.001. The next model testing the effect of loss of pleasure on common EF had good fit, *χ^2^*_(118)_ = 505.647, *p* < 0.001, CFI = 0.977, SRMR = 0.043. The loss of pleasure factor was not significantly associated with common EF, *β* = −0.037, *p* = 0.587. The model evaluating the effect of loss of interest on common EF had good fit, *χ^2^*_(43)_ = 123.804, *p* < 0.001, CFI = 0.985, SRMR = 0.037. The loss of interest factor had a significant negative association with common EF, *β* = −0.143, *p* = 0.046. The final model tested the effect of the mania factor on common EF. This model had good fit, *χ^2^*_(64)_ = 112.607, *p* < 0.001, CFI = 0.988, SRMR = 0.038. The mania factor significantly related to lower Common EF, *β* = −0.231, *p* = 0.005.

#### Exploratory Symptom Factors and Common EF

A structural equation model was tested in which common EF was regressed on the general and specific factors from the 5-factor exploratory bifactor model (Figure 5C). This model fit well, *χ^2^*_(1713)_ = 2,392.579, *p* < 0.001, CFI = 0.983, SRMR = 0.036. The exploratory general factor, on which all items significantly loaded, was significantly related to common EF, *β* = −0.243, *p* = 0.001, with a small effect size. Exploratory Specific Factor 2 (primarily characterized by items related to physiological arousal) had a small but significant association with Common EF, *β* = −0.176, *p* = 0.037. However, the association between Exploratory Specific Factor 2 and common EF fell to the level of a trend after FDR correction (*q* = 0.08). The association between the exploratory general factor and Common EF remained significant after FDR correction (*q* = 0.005). This model accounted for 10.4% of the Common EF factor variance.

## Discussion

This study examined associations between common EF and general or specific dimensions of mood symptoms, in adolescents and young adults with high variance in symptom severity. In both confirmatory and exploratory bifactor models, higher levels of a general depression factor capturing shared variance across mood symptom dimensions was significantly associated with lower executive function across tasks. In addition, higher levels of a specific factor primarily reflecting physiological arousal were significantly associated with lower common EF, in both confirmatory and exploratory analyses. Altogether, results indicate that general deficits in goal-directed behavior are broadly associated with features shared across mood symptom dimensions, but also specifically associated with physiological arousal.

The association between higher levels of general depression and lower common EF (identified in both confirmatory and exploratory factor analyses) aligns with previous studies, which have found associations between the shared variance across depression and anxiety symptoms and common EF at both the behavioral and genetic level (Hatoum et al., 2018; Gustavson et al., 2019; Snyder et al., 2019). In our confirmatory factor analyses, the general depression factor was anchored by the MASQ general distress—depression and loss of interest items, which together reflect the cardinal symptoms of depression (sad mood and anhedonia). In our exploratory factor analysis, the General Depression factor showed a similar pattern of loadings, with the highest loadings from the MASQ general distress—depression and loss of interest scales. The association between general depression and common EF can be interpreted in several ways. Lower overall EF may represent vulnerability to experience multiple types of (often, co-occurring) symptoms that are together captured by the general depression factor, perhaps especially under conditions of stress (Snyder et al., 2019; Peterson et al., 2021). Alternatively, the negative affective state captured in the general depression factor may have an impairing effect on common EF. For example, greater distress or negative effect may exacerbate the use of maladaptive response styles such as rumination (Nolen-Hoeksema et al., 1999; Calvete et al., 2015), which in turn can have detrimental effects on task performance (Watkins and Brown, 2002). Recent work supports the existence of bidirectional pathways, in which EF may act as a vulnerability factor for psychopathology, and psychopathology may in turn exacerbate dysfunctions in EF (Romer and Pizzagalli, 2021; Zainal and Newman, 2021). Additional longitudinal studies extending this work to other age groups and further exploring potential moderators and mediators may help illuminate the mechanistic pathways underlying this association.

These results highlight a significant association between common EF and general depression in adolescence and young adulthood. Prior research has supported such an association in children, adolescents, and adults (Caspi et al., 2014; Martel et al., 2017; Snyder et al., 2019), suggesting that this relationship is present throughout the lifespan. However, adolescence may be a particularly salient developmental period for evaluating the association between common EF and mood pathology, given that this is a time of profound EF development as well as a period of heightened risk for mood problems. An interesting direction for this work will be investigating the interactions between cognitive development and mood pathology. For example, one study examining adolescents and young adults found that the association between common EF and general psychopathology increased with age (Snyder et al., 2019); another study identified a similar pattern in which the association between mania and common EF strengthened during this developmental period (Kaiser et al., 2021). While outside the scope of the current study, examination of the dual and potentially bidirectional effects of cognitive development and psychopathology warrants continued exploration, especially in longitudinal samples.

Consistent with prior research (Warren et al., 2021), we also identified a significant association between a symptom dimension of physiological arousal and lower common EF. In the confirmatory model, this factor was restricted to canonical anxious arousal items (which primarily reflect sympathetic nervous system arousal). In the exploratory model, a specific factor emerged that predominantly captured items from the canonical anxious arousal scale (e.g., “Muscles twitched or trembled”). However, it also captured small but significant loadings from other items reflecting greater positive affect and lower negative affect. This pattern could reflect the fact that the general factor already captures negatively valenced forms of arousal, and what remains simply reflects physiological arousal. Across both confirmatory and exploratory factor analyses, lower physiological arousal was associated with better common EF, suggesting that EF may serve as a protective factor that helps individuals modulate their level of arousal through its role in self-regulation (Nigg, 2017), or that excessively high arousal disrupts task performance (Yerkes and Dodson, 1908). Notably, the modification indices also suggested a potential correlation between the mania-specific and arousal-specific factors. While this correlation was not added to retain orthogonality of the factors, it may reflect the fact that heightened sympathetic nervous system arousal often accompanies mania (Henry et al., 2010). Thus, physiological arousal in this sample may be occurring in the context of mania as well as anxiety. Altogether, the association between physiological arousal and common EF suggests that this symptom dimension may relate to common EF through a separate mechanistic pathway from general depression.

Other specific factors were not associated with common EF in either the confirmatory or exploratory bifactor models. However, all symptom dimensions were individually associated with common EF when modeled separately—with the exception of the standalone loss of pleasure factor. One interpretation is that low positive affect does not interfere with EF; some research has suggested that positive mood can in fact impair EF performance (Mitchell and Phillips, 2007). Alternately, common EF may primarily protect individuals from negative affect without necessarily boosting positive affect (e.g., Short et al., 2016). Null effects could also originate from methodological differences: the canonical loss of pleasure scale is the only measure in the MASQ that consists entirely of reverse-coded statements; thus, its specific variance may in part reflect method variance. Finally, while this scale may capture the presence or absence of positive effect, it might not effectively capture the *inability* to experience the pleasure that defines consummatory anhedonia (Treadway and Zald, 2011). In contrast, the standalone loss of interest factor was individually associated with EF, highlighting the importance of carefully decomposing the symptom dimension of anhedonia.

Overall, the confirmatory and exploratory analyses revealed consistent patterns of correlations between common EF and symptom dimensions: both identified significant associations between common EF and factors reflecting general depression and physiological arousal. However, an additional comment is warranted regarding differences in the symptom dimensions that emerged from these two modeling approaches. We note that while both approaches yielded conceptually similar symptom structure, there were also key differences: e.g., the exploratory model yielded four specific factors, whereas the confirmatory model fit best with three specific factors. Moreover, the nature of the factors derived from each analysis differed in several ways. For example, in the confirmatory model, the canonical loss of interest items were subsumed by the general factor, which aligns with prior models also using the MASQ (Banich et al., 2020). However, in the exploratory model, a specific factor emerged which included a number of items from the loss of interest scale, but also items from the canonical loss of pleasure, general distress—depression, and anxious arousal scales (Specific Factor 1). This exploratory specific factor seemed to capture a mixture of low motivation, low positive effect, and sad mood, but also had negative associations with symptoms of fatigue and physiological arousal. These patterns point to a divergence between the MASQ conceptualization of the loss of interest dimension, and how a data-driven approach clusters these items. Given the exploratory nature of these analyses, future studies should examine the replicability of these dimensions in other samples. Alternative measures of anhedonia may also help identify separable components of this construct.

Several limitations of this study should be addressed. First, we utilized a cross-sectional design, which does not allow for clarification of the temporal relationship between common EF and various symptom dimensions. Future studies examining changes in the associations between general and specific mood symptoms and common EF over time, or comparing high-risk, currently symptomatic, and remitted individuals, may help illuminate the causal mechanisms underlying these relationships. In addition, this study focused on mood disorders rather than other forms of psychopathology, leaving it unknown whether other specific symptom dimensions have similar or different associations with common EF. This approach allowed a targeted investigation of mood symptom dimensions, but future research may extend to additional clinical samples. For example, studies that include more individuals with primary anxiety disorders, as well as mood disorders, may be better suited to looking at internalizing symptoms more generally. Additionally, while our sample was transdiagnostic across mood disorders, there was a higher proportion of individuals with unipolar vs. bipolar forms of depression (Table 2). However, the sample still exhibited a significant variance in manic symptoms, consistent with prior studies showing that individuals with unipolar disorders frequently experience subclinical manic symptoms (Carlson and Kashani, 1988; McIntyre et al., 2015). A valuable next step would be to explore these models in larger samples of individuals with bipolar disorders. These models could be augmented in a variety of ways, such as by expanding the EF model, incorporating additional symptom dimensions, or controlling for other variables that may influence these relationships. Here, we chose to focus on common EF based on prior research suggesting that this dimension is most consistently linked with psychopathology (Hatoum et al., 2018; Snyder et al., 2019). However, future studies utilizing a larger suite of EF tasks may wish to extract specific EF factors, including but not limited to shifting and updating, and examine their relationship with various symptoms of psychopathology. Additionally, a next step in testing this model will be to incorporate objective measures (e.g., of physiological arousal) and compare them with subjective self-report measures of symptom dimensions. In this study, we identified an association between higher self-reported sympathetic arousal and lower common EF; however, prior work has suggested that self-report and physiological measures of arousal can diverge (Miers et al., 2011). Therefore, a multi-modal evaluation of these constructs may provide a more nuanced understanding of psychiatric health and cognitive functioning. Future analyses may also benefit from controlling for factors such as processing speed, since prior work has shown that processing speed is correlated with, but separable from, Common EF (Friedman et al., 2008). Finally, the model could be modified by using a different reference domain for the general factor. For example, we chose in our confirmatory factor analysis to anchor the general factor with items from the canonical general distress—depression scale, based on the predominance of individuals with mood disorders in our sample. However, the most appropriate reference domain for a general factor in a bifactor S-1 may vary depending on the research question, study sample, and theoretical interpretations of the meaning of the general factor.

In sum, this study identified significant associations between lower executive functioning ability (common EF) and symptom dimensions representing general depression and physiological arousal. These results suggest that general depressive symptoms and physiological arousal may be associated with common EF *via* different mechanisms, and highlight the value of distinguishing between the shared and specific features of symptom dimensions.

## Data Availability Statement

The datasets presented in this article are not readily available because evaluation of other experimental hypotheses are ongoing in this dataset. Data will be available upon request after other a priori analyses have been completed. Requests to access the datasets should be directed to RK, roselinde.kaiser@colorado.edu.

## Ethics Statement

The studies involving human participants were reviewed and approved by the University of Colorado Boulder Institutional Review Board and the University of Los Angeles California Institutional Review Board. Written informed consent to participate in this study was obtained from legal adults (ages 18 and older) and parental consent and child assent were obtained from legal minors (ages 17 and younger).

The studies involving human participants were reviewed and approved by the University of Colorado Boulder Institutional Review Board and the University of Los Angeles California Institutional Review Board. Written informed consent to participate in this study was provided by the participants’ legal guardian/next of kin.

## Author Contributions

RK and EP developed the study concept and design. EP, CN, BR, CH, CS, LO, SG, JK, JF, CR, AG, and SS performed data collection. EP, CN, BR, CH, and CS performed clinical evaluation, under RK’s supervision. DM provided consultation on recruitment and clinical evaluation. EP performed analyses with assistance from RK, NF, and HS. The manuscript was drafted by EP and all other co-authors provided critical revisions. All authors contributed to the article and approved the submitted version.

## Conflict of Interest

The authors declare that the research was conducted in the absence of any commercial or financial relationships that could be construed as a potential conflict of interest.

## Publisher’s Note

All claims expressed in this article are solely those of the authors and do not necessarily represent those of their affiliated organizations, or those of the publisher, the editors and the reviewers. Any product that may be evaluated in this article, or claim that may be made by its manufacturer, is not guaranteed or endorsed by the publisher.

## Footnotes

^1^We note that due to researcher error, the instructional text preceding the GBI items was inconsistent across sites (UCLA, CU Boulder) in directing the participant to consider the past-week timeframe when reporting on symptoms of mania. Sensitivity analyses were performed to ensure that this error did not engender site differences that influenced results, including testing for site differences in symptom factor structure and associations between symptoms and Common EF.
